# Indoor Positioning System Based on a PSD Detector, Precise Positioning of Agents in Motion Using AoA Techniques

**DOI:** 10.3390/s17092124

**Published:** 2017-09-15

**Authors:** David Rodríguez-Navarro, José Luis Lázaro-Galilea, Álvaro De-La-Llana-Calvo, Ignacio Bravo-Muñoz, Alfredo Gardel-Vicente, Georgios Tsirigotis, Juan Iglesias-Miguel

**Affiliations:** 1 Department of Electronics, University of Alcalá, Alcalá de Henares, 28801 Madrid, Spain; david.rodriguezn@edu.uah.es (D.R.-N.); alvaro.llana@uah.es (A.D.-L.-L.-C.); ignacio.bravo@uah.es (I.B.-M.); alfredo.gardel@uah.es (A.G.-V.); juan.iglesiasm@edu.uah.es (J.I.-M.); 2 Computer and Informatics Engineering Department, Eastern Macedonia and Thrace Institute of Technology, 65404 Kavala, Greece; tsirigo@teiemt.gr

**Keywords:** indoor positioning system, PSD, optical signal, AoA, multi-agent localization

## Abstract

Here, we present an indoor positioning system (IPS) for detecting mobile agents based on a single Position Sensitive Device sensor (PSD) sited in the environment and InfraRed Emitter Diode (IRED) located on mobile agents. The main goal of the work is to develop an alternative IPS to other sensing technologies, cheaper, easier to install and with a low computational load to obtain a high rate of measurements per second. The proposed IPS has the capacity to accurately determine 3D position from the angle of arrival (AoA) of the signal received at the PSD sensor. In this first approach to the method, the agents are considered to move along a plane. We propose two alternatives for determining position: in one, tones are emitted on a frequency unique to each transmitter, while in the other, sequences are emitted.The paper proposes and set up a very simple and easy to deploy system capable of performing 3D positioning with a single analog sensor, obtaining a high accurate positioning and a reduced execution time for the signal processing. The low computational load of the IPS makes it possible to obtain a very high position update rate (more than 100 times per second), yielding millimetric accuracies.

## 1. Introduction

Indoor positioning systems (IPS) use a wide range of technologies [1], each of which presents different characteristics tailored to different applications, with varying degrees of success. The choice of technology is determined by different requirements, such as cost, maintenance, privacy, accuracy, position update speed, cover, consumption and infrastructure. However, it should be noted that all these technologies can be complementary.

IPS have multiple applications that range from providing support for people with special needs [2] to tracking users in large areas [3], guiding people at train stations, airports [4], museums [5], supermarkets [6], bookstores [7] and car parks [8] and providing security at major events, hospitals [9], prisons, underground stations, airports and on trains.

In the industrial field, IPS applications are used in smart factories [10] that deploy autonomous robots that move and cooperate with each other to perform various tasks. One example of this type of application is presented in [11], which used an autonomous robot to move objects within an environment.

The currently available IPS technologies are presented in [1], giving examples of each together with their characteristics. The technologies of interest in this study were those systems that yield centimetric accuracies, namely vision, magnetic, ultrasound and infrared systems. An example of a vision-based IPS is presented in [12], in which a camera was sited on the ceiling of the environment and artificial marks were placed on the agents, achieving accuracies of 1 cm and 5° in the orientation of the agent.

Another technology of interest is ultrasound. This is widely used due to the low cost of the infrastructure while yielding good accuracy, and one of the best known IPS is the Cricket system [13], which is based on calculating the time of flight from transmitters located in the environment to the receiver incorporated in the agent, synchronising clocks by means of an RF (Radio Frequency) signal. In [14], ultrasound was used to perform measurements of the Time Difference of Arrival (TDoA) in order to determine position, emitting different sequences for each transmitter located in the environment and equipping the mobile agent with a receiver.

Another technology of great interest is RF. The RF-based systems have several advantages over other systems such as using infrastructure already installed (e.g., WLAN (Wireless Local Area Network) access points) in large spaces such as shopping malls, airports, etc., which makes it a low cost system, with a coverage distance around tens of meters, besides that they do not required a direct line of sight path (LOS) between emitter and receiver, therefore they have longer coverage even in the presence of obstacles. On the other hand, RF-based systems have a reduced accuracy up to several tens of centimeters, even reaching meters of error according to the distances between emitter and receivers, and presence of obstacles (furniture, building structure, people, etc.). A review of the different positioning methods used by RF-based systems is done in [15] and more recent examples can be found in [16] where the position determination of the target is done by means of geometrical methods achieving errors of several decimeters, being a simple and robust algorithm to be used in noisy environments. In [17], they develop an IPS merging RF and ultrasound technologies, using RF to obtain the starting point of the tracking and the ultrasound system for later displacements obtaining higher accuracy.

There are a few and also old research works that use PSD (Position Sensitive Detector) sensors for the indoor positioning of mobile agents, for example [18,19]. In [18], the authors propose a PSD-based IPS placed on a height of 2.5 m making use of a Kalman filter to track a mobile robot that moves at a constant speed on a ground floor. The location results give a maximum error of 8.97 cm and average error of 1.97 cm, in a coverage area of 3 × 3 m${}^{2}$. The maximum error comes from the location of the robot in the periphery of the monitored area.

On the other hand, Ref. [19] proposes a multiple PSD system located around the surveillance area where a mobile robot with an emitter moves freely. The positioning algorithm is done by means of trilateration, obtaining the distances from each PSD and the robot through stereoscopic measurements. The results achieved in the test indicate that the error in the distance measurements increase linearly from 70 cm to 440 cm showing a nonlinear behavior outside of that distance boundary.

Visible or infrared light-based IPS are possibly the least developed to date. Related to these approaches, the other potential function of LED (Light-Emitting Diode) light in the context of VLC (Visible Light Communication) can be wireless communication and positioning, which make them an attractive research topic. The duplicate use of LEDs for lighting eliminates the cost of installing a positioning system based on VLC. Furthermore, the absence of electromagnetic interferences makes the use of LEDs particularly interesting; positioning based on VLC can be used as an indoor navigation system for location tracking, finding objects, controlling the movement of agents, etc.

Interesting works for the reader such as [20] proposes a design for an indoor positioning system using LEDs, an image sensor (IS) and an accelerometer from mobile devices. The proposed scheme consists of four LEDs mounted on the ceiling transmitting their own three-dimensional world coordinates and an IS at an unknown position receiving and demodulating the signals. Based on the 3D world coordinates and the 2D image coordinate of LEDs, the position of the mobile device is determined. To further improve the accuracy, also propose a mechanism to reduce the image sensor noise. With the assumption that the IS coordinate space is parallel to the world reference plane, they evaluate the accuracy obtaining values below 10 cm. They also make a comparison with other state-of-the-art techniques such as [21,22] showing the reaching of similar accuracy results. Another example is presented in [23], in which a visible light-based IPS was designed using multiple photodiodes in the environment and the received signal strength (RSS) to determine the position of the mobile agent, obtaining mean error of 7 cm in a range of 2 m. The main problem encountered was the low SNR (Signal-Noise Ratio) as distance and angles increase. Work in [24] proposes an indoor VLC and positioning system using the orthogonal frequency division multiplexing access scheme (OFDMA), in which the signals transmitted by LEDs are encoded with allocated subcarrier, respectively, and the receiver recovers all transmitted signals using a discrete Fourier transformation (DFT) operation. The feasibility of the scheme is demonstrated in a room of size 20 × 20 × 15 cm${}^{3}$. They show that the proposed scheme offers a mean positioning error of 1.68 cm, overcoming 2 cm of maximum error.

The work in [25] proposes a system based on LED-beacons and an IR-camera (InfraRed-camera). The modulation and detection technologies used are similar to those of VLC, not optimized for bandwidth but for an optimal detection of the LED beacon. They obtain a 5% of false positives and false negatives in the location of the LED beacons.

In another similar work [26], the authors also use four LEDs and IR-camera. The paper proposes a fast coding/decoding scheme to be able to use low-cost artificial vision devices (e.g., smartphones).

The research work [27] proposes a different system, based on emitting IRED (InfraRed Emitting Diode) beacons from the mobile agents and deploying an array of photodiodes as detectors. The 3D positioning is obtained by measuring the angle between the emitter and several photodiodes receivers. The tests were done in a coverage area of 7 m × 2 m obtaining errors up to 0.7 m . This system is more expensive and difficult to install, calibrate and deploy than the system we propose in our paper based on a single PSD sensor.

The main drawback of these technologies concerns the multipath (MP) by which the signal reaches the receivers. Looking solely at optical signals, the model of near-infrared (NIR) signal reflection reported in [28] enabled us to model and analyze how this affects AoA (Angle of Arrival) and DPoA (Difference Phase of Arrival) measurement techniques and take this into account in our study.

Here, we present an indoor positioning system (IPS) with the capacity to accurately determine the position of mobile agents based on optical signals and a PSD sensor. The computational load of the system and methods proposed is very low, making it possible to obtain a very high position update rate. In this first report on the method, we focus on describing the proposals and evaluating the positioning methods.

Based on [29], which describes the sources of electrical errors in a PSD sensor system and how to correct and mitigate the effects of these sources, and [30], which describes a geometric model of a PSD sensor-optics system and a calibration process to obtain intrinsic parameters, we designed a system for determining the 3D position of mobile agents using the angle of arrival (AoA).

## 2. Background

To determine 3D position from the angle of arrival, it is necessary to perform an electrical calibration as described in [29], and a geometric calibration as described in [30]. In [29], the authors modeled signal conditioning and sources of error (gain factor imbalances, temperature variations, signal noise and quantification noise). The main errors arose from signal noise and gain imbalances in the PSD sensor channels, which could be substantially mitigated by using digital filters and performing electrical calibration.

It should be noted that the intrinsic parameters of the set composed by the PSD + lens when obtaining the positioning of some mobile agent, as developed in [29,30], must be perfectly modeled and calibrated; otherwise, the errors are huge, turning useless the system.

Once electrical calibration has been performed, the final equations for calculating the point of impact are given by Equations (1) and (2):

(1) $$x=\frac{L_{x}}{2}\frac{\left(V_{X2}+V_{Y1}\right)-\left(V_{X1}+V_{Y2}\right)}{V_{X1}+V_{X2}+V_{Y1}+V_{Y2}},$$

(2) $$y=\frac{L_{y}}{2}\frac{\left(V_{X2}+V_{Y2}\right)-\left(V_{X1}+V_{Y1}\right)}{V_{X1}+V_{X2}+V_{Y1}+V_{Y2}}$$

where $V_{xi,yi}$ are the amplifier stage output signals, and $L_{x}$ and $L_{y}$ are PSD sensor size.

**Figure 1:**
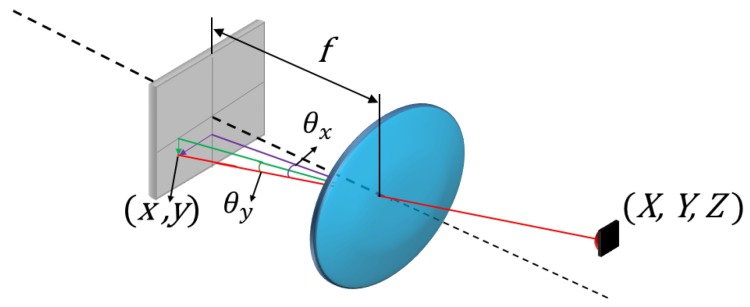
Receiver based on Position Sensitive Device sensor (PSD) and lens.

Meanwhile, geometric calibration is based on obtaining the parameters that model the receiver system (Figure 1) in order to calculate the angle of arrival.

In Figure 1, (x,y) represent the points in the PSD sensor, ($C_{x},C_{y}$) is the optical center, *f* is the focal length, (X,Y,Z) are the points in the transmitter in the environment and ($\theta_{x},\theta_{y}$) are the angles of arrival, using Equations (3) and (4) to calculate the angles:

(3) $$\theta_{x}=\tan^{-1}\left(\frac{x}{f}\right),$$

(4) $$\theta_{y}=\tan^{-1}\left(\frac{y}{f}\right).$$

As can be seen, the field of vision is related to the size of the PSD sensor and the focal length of the lens, according to Equation (5):

(5) $$\mathrm{FoV}=2\tan^{-1}\left(\frac{d}{2f}\right),$$

where *d* is the diagonal of the sensor and *f* is the focal length of the lens.

The optical system must meet certain requirements. These include the balance between lens size and the energy it will receive, and, therefore, the SNR, and the compromise between focal length and the FoV (Field of View) that the sensor covers. It is also necessary to consider aspects of manufacture, since the longer the focal length, the larger the lens diameter (or the lens would be too thick), and, consequently, the FoV is reduced and may not reach the desired scope.

The solution to this problem is to use an optical group composed of various lenses, which reduces transmittance because each lens reflects part of the light; however, this means that, in systems where the received signal strength is low, the required SNR may not be achieved.

**Figure 2:**
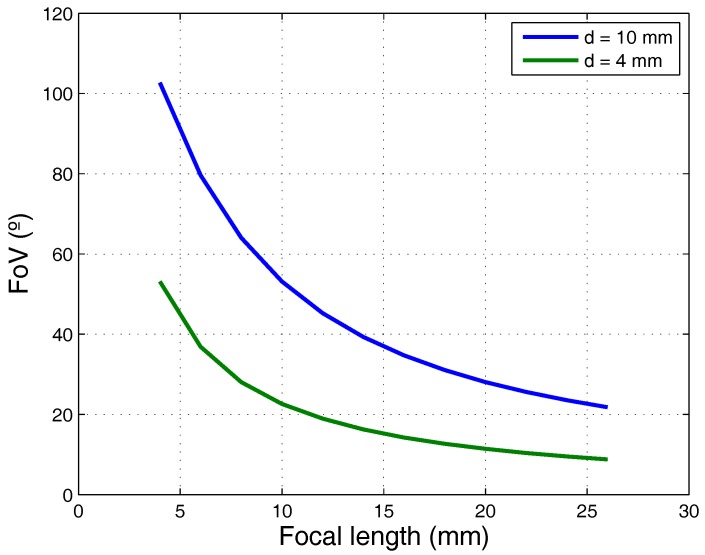
FoV depending on focal length and PSD sensor size.

Figure 2 gives an example of the field of view with two sensor sizes and various focal lengths.

Given the above background, we describe below the PSD sensor based positioning strategies to perform a high number of measurements per second and determine the position of multiple agents simultaneously without losing location accuracy, highlighting the simplicity of these methods.

## 3. Proposed Positioning Method

**Figure 3:**
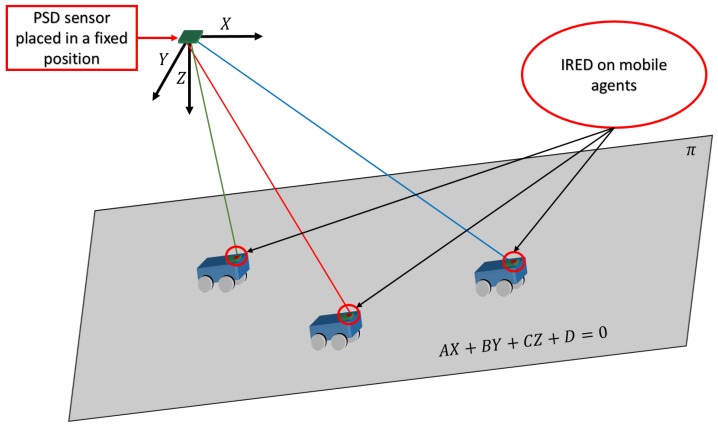
Diagram of the proposed IPS.

Here, we describe a method for determining mobile positions using a single receiver and assuming that the mobile robot moves along a plane. Under these conditions, the geometry of the plane and the direction vector of the agents relative to the receiver must be determined to obtain the intersection of plane and direction, which gives the 3D position. Figure 3 shows a diagram of the proposed IPS; the detector is located in the environment and the mobile agents equipped with IRED transmitters move along a plane.

In Figure 3, (X,Y,Z) are the coordinates for points in the environment with reference to the receiver and (A,B,C,D) are the parameters that model the plane.

The proposed positioning method is based on three steps:

- determining the equation for the plane of movement of the agents;
- determining the angle of the agent with respect to the PSD sensor;
- obtaining the 3D positions.

The equation for the plane is obtained in an offline procedure; the vector direction of the agents is obtained from the point of impact on the PSD sensor of the signal emitted using Equations (3) and (4) and the 3D position is obtained from the intersection of the vector direction and plane.

To determine the position, two alternatives are proposed with respect to the signals used; the first is based on sinusoidal signals (different frequencies for different agents, proposed when commencing the study) and the second on emitting sequences and correlating the signal received with the replicas of the same.

### 3.1. Calibration of the Plane

To calculate the plane along which the mobile robot moves, it is necessary to determine the 3D position of three or more points on that plane, relative to the receiver. We used a calibration template to obtain the rotation matrix and the translation vector between the template and the receiver, and subsequently to obtain the plane parameters. This template consists of transmitters located in the environment at five or more points with known relative positions that cover the largest possible area of the receivers FoV. These relative positions and their images in the sensor are used to obtain a projection matrix; then, the rotation and translation matrices are calculated followed by the plane parameters, as described below.

- Projection matrix (homography): where ($X_{t},Y_{t}$) are the coordinates of the points of the two-dimensional template relative to any point in the template, and (x,y) represent the projection of these points onto the receiver. Singular value decomposition of the matrix system (6) yields the projection matrix elements $m_{ij}$, as explained in [31]:
  (6) $$\left(\begin{array}{ccccccccc} X_{t} & Y_{t} & 1 & 0 & 0 & 0 & -x\cdot X_{t} & -x\cdot Y_{t} & -x\\ 0 & 0 & 0 & X_{t} & Y_{t} & 1 & -y\cdot X_{t} & -y\cdot Y_{t} & -y \end{array}\right)\left(\begin{array}{c} m_{11}\\ m_{12}\\ \vdots \\ m_{33} \end{array}\right)=0.$$
- Rotation matrix and translation vector: once the projection matrix has been obtained, the rotation matrix and translation vector parameters are obtained using Equations (7)–(10):
  (7) $$r_{1}=\lambda A^{-1}m_{1},$$
  (8) $$r_{2}=\lambda A^{-1}m_{2},$$
  (9) $$r_{3}=r_{1}\times r_{2},$$
  (10) $$t=\lambda A^{-1}m_{3},$$
  where $\lambda =\left|\left|\frac{1}{A^{-1}m_{1}}\right|\right|$, *A* is the matrix of the intrinsic parameters obtained from the geometric calibration, and $m_{1},m_{2}$, and $m_{3}$ are the column vectors of the projection matrix. Next, the rotation matrix and translation vector are used to calculate the 3D coordinates of the template points with respect to the receiver:
  (11) $$\left(\begin{array}{c} X\\ Y\\ Z \end{array}\right)=\left[\begin{array}{ccc} r_{1} & r_{2} & t \end{array}\right]\left(\begin{array}{c} X_{t}\\ Y_{t}\\ 1 \end{array}\right).$$
- Obtaining the plane parameters: the final task is to obtain the values that model the plane. Using three or more of the previously obtained points that do not belong to the same line, resolving the system (12) by SVD, the plane parameters are obtained:
  (12) $$\left(\begin{array}{cccc} x_{1} & y_{1} & z_{1} & 1\\ x_{2} & y_{2} & z_{2} & 1\\ \vdots & \vdots & \vdots & \vdots \\ x_{n} & y_{n} & z_{n} & 1 \end{array}\right)\left(\begin{array}{c} A\\ B\\ C\\ D \end{array}\right)=0.$$

### 3.2. Calculating the Point of Impact

#### 3.2.1. Using Sinusoidal Signals

In the case of using sinusoidal signals, the number of different frequencies (agents) that can be used in the future when operating with more than one transmitter will depend on the system BW (BandWidth). In any event, using one or more tones, the position is obtained by calculating the RMS (Root Mean Square) value of the output signals from the four PSD sensor channels. The higher the frequency employed, the lower the acquisition time required to perform good tracking.

Once the emitted frequency (or frequencies) is known, a higher SNR is obtained by using an IIR (Infinite Impulse Response) filter, which has a smaller number of coefficients and thus implies a lower computational load. Narrower filters can be used with fewer coefficients (with several agents, it will be necessary to filter each tone to isolate their signals). However, note that an IIR filter may oscillate. Equation (13) gives the general equation.

(13) $$\begin{array}{cc} y[n] & =a_{0}x\left[n\right]+a_{1}x\left[n-1\right]+a_{2}x\left[n-2\right]+\cdots +a_{N}x\left[n-N\right]-\\ & \;-b_{1}y\left[n-1\right]-b_{2}y\left[n-2\right]-\cdots -b_{M}y\left[n-M\right], \end{array}$$

where *x* is the digitised input signal, *y* is the signal at the filter output, and $a_{i}$ and $b_{k}$ are the filter coefficients, where $i=\left\{0,1,\ldots ,N\right\}$ and $k=\left\{0,1,\ldots ,M\right\}$.

Once the four PSD sensor signals have been filtered, their RMS values are calculated and Equations (1) and (2) are then used to calculate the points of impact.

#### 3.2.2. Using Sequences

The other alternative consists of emitting different sequences and correlating reception with their replicas; the advantage of this method is that agents can easily be added and the SNR can be improved using longer sequences. However, long sequences lead to increased acquisition times and slower position update rates.

As regards the type of sequence to use, for our initial tests, we selected GOLD sequences modulated by BPSK (Binary Phase Shift Keying) with a frequency equal to the chirp frequency. To calculate the point of impact, the PSD sensor output signals are correlated with the replicas to obtain a peak value for each channel, and these four values are used in Equations (1) and (2) to calculate the point of impact, where V represents the correlation values. Interference errors may occur if different sequences are used simultaneously, but these can be mitigated or corrected by algorithms, as detailed in [32].

### 3.3. Determining the 3D Position of the Mobile Robot

Once the displacement plane, the angle of arrival and the point of impact on the PSD sensor are known, the position can be deduced.

The equation for the plane in parametric form is:

(14) AX+BY+CZ+D=0,

and the equation for the line of arrival of the signal to the receiver is:

(15) $$X_{i}=\tan\;\theta_{x_{i}}Z_{i},$$

(16) $$Y_{i}=\tan\;\theta_{y_{i}}Z_{i},$$

where *i* represents each transmitter, ($\theta_{x},\theta_{y}$) are the angles of arrival and (X,Y,Z) are the 3D coordinates with reference to the receiver.

Substituting Equations (15) and (16) in Equation (14) and resolving *Z*, we have:

(17) $$Z_{i}=-\frac{D}{A\tan\;\theta_{x_{i}}+B\tan\;\theta_{y_{i}}+C}.$$

Thus, replacing *Z* in (15) and (16) yields *X* and *Y*, thus obtaining the 3D position of the mobile robots.

## 4. Error Sensitivity

Error in position determination is given by error when obtaining the plane parameters (due to errors in determining the points of the template) and error when determining the angle of arrival.

### 4.1. Z Sensitivity with Respect to the Plane Parameters

The sensitivity of depth determination with respect to the plane parameters is obtained by derivating the A,B,C and *D* parameters from Equation (17). This sensitivity depends on the location of the receiver relative to the plane of movement of the mobile agent.

To provide an example of sensitivity when determining the *Z* coordinate due to errors in the calculation of the plane parameters, we will assume two situations: (a) with a plane coplanar to the PSD sensor, and (b) with a 15° incline. Because template parameter sensitivity depends on template size, we have assumed a receiver with a 30° field of vision sited 4 m high and covering 1800 mm. Table 1 shows the 3D coordinates used for the calibration template and the parameters of both planes are those in Table 2.

**Figure 4:**
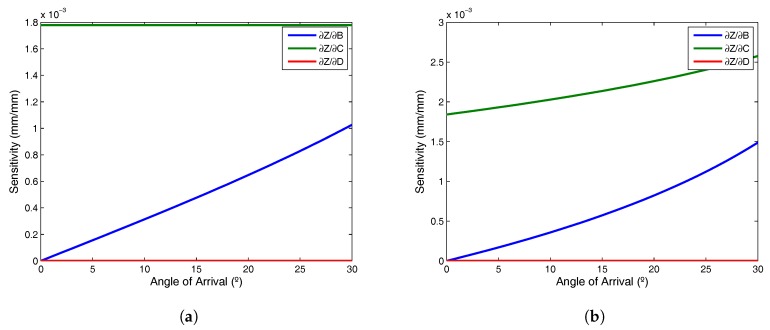
Z sensitivity with respect to the plane parameters, (**a**) coplanar plane; (**b**) inclined plane.

Figure 4a,b show the sensitivity, expressed as deviation in determining *Z* coordinate (mm) per each mm of error committed in determining the parameters of the plane (Table 2).

**Figure 5:**
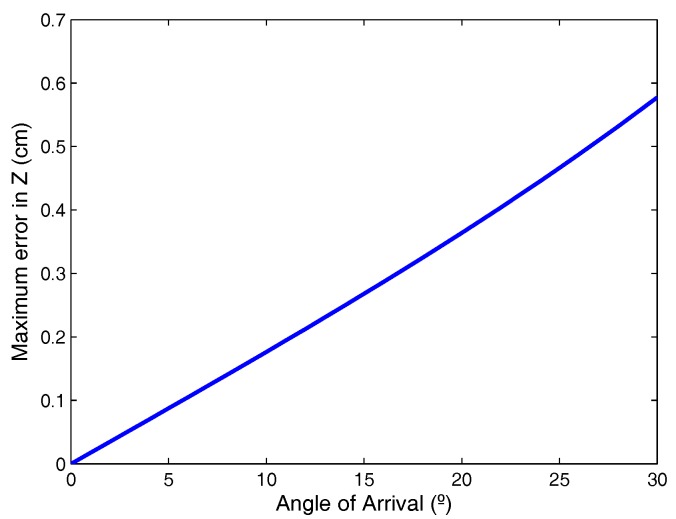
Absolute error in Z due to errors in calculation of the plane.

Figure 5 shows the maximum absolute error in the calculation of the *Z* coordinate, depending on the angle of arrival, when the error for template points is 1 cm.

As can be seen, small errors in the plane calibration template can give rise to not negligible errors in the calculation of *Z*; however, such errors can easily be minimized if the procedure is performed carefully.

### 4.2. Z Sensitivity with Respect to the Angles of Arrival

Another contributory factor to error when determining the 3D position of the mobile robot is the error committed when calculating the angle of arrival. In this case, the uncertainty of the depth error is obtained by derivating the incidence angles ($\theta_{x},\theta_{y}$) from (17).

**Figure 6:**
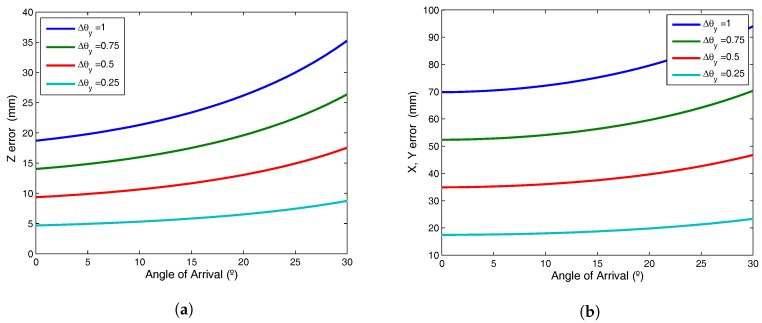
Errors due to the error in determining the angles of arrival; (**a**) error in *Z* coordinate; (**b**) errors in *X* and *Y* coordinates.

Figure 6a shows the error in *Z* for different errors in the angle of arrival when the plane is inclined 15${}^{\circ }$, since the error is 0 in the case of the coplanar plane and the errors in *X* and *Y* coordinates due to the AoA error is shown in Figure 6b.

As can be seen, small errors in the calibration template and determination of the angle of arrival can give rise to substantial error in the calculation of *Z* and also generates greater error in the *X* and *Y* coordinates than in *Z*.

This section has demonstrated that small errors in calibration of the plane of movement of the mobile agents and calculation of the angle of arrival may lead to decimetric errors. Below, we report the results of experimental tests, indicating the magnitudes of error in the plane parameters and in determination of the angle of arrival.

## 5. Empirical Results

**Figure 7:**
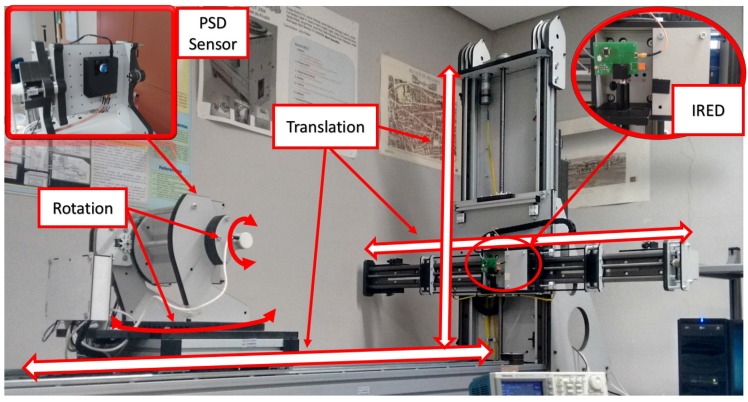
Automated support for determining angle error.

In this section, we report the results of three experimental tests. In the first, we used the automated support shown in Figure 7 to quantify the error in determining the angle of arrival. The second test consisted of determining the 3D position of an IR transmitter in static situations, while the third test involved a moving agent (in both cases using a sinusoidal signal and a sequence). The results were compared with those obtained with a vision-based IPS.

**Figure 8:**
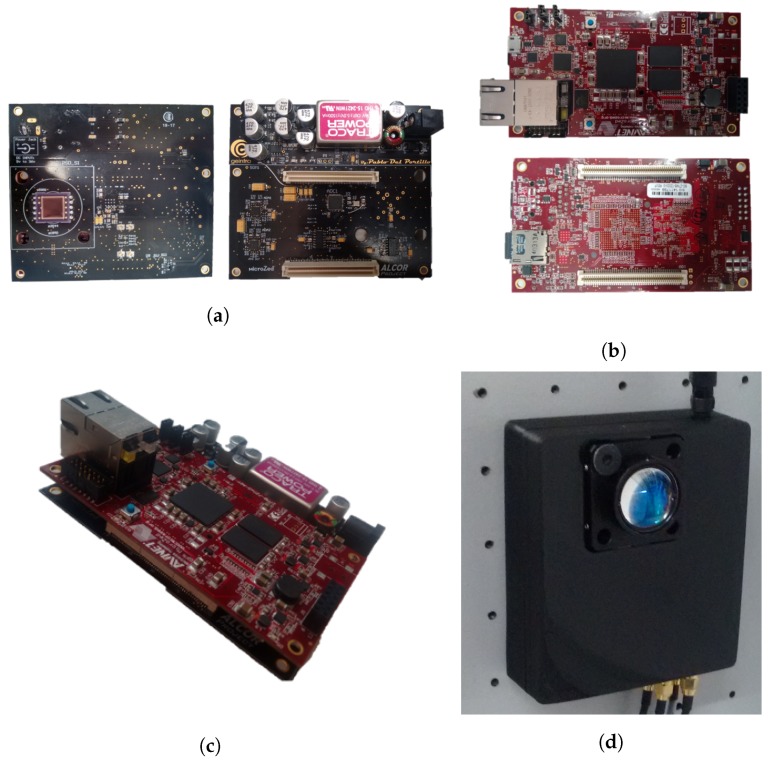
Proposed detector system; (**a**) board for signal conditioning; (**b**) MicroZed™; (**c**) connected boards; (**d**) final enclosed system.

For the tests, we used a Hamamatsu (Hamamatsu City, Shizuoka Pref., Japan) S5991-01 PSD sensor and a 1-inch diameter, 16 mm ± 8% focal length lens and an IR emitter Osram (Munich, Germany) SFH 4233, selected because it is a high power emitter with a Lambertian pattern and low rise and fall times. Two different systems have been used to carry out the tests. For the initial laboratory tests done with the workbench, we have used the following components: a data acquisition board GAGE (Lockport, IL, USA) CS8284 configured with a sampling frequency of 10 MS/s, 12-bit resolution and input voltage range from 100 mV to 5 V. To process the acquired data we have used a PC (CPU Intel^®^ Core™ i5-660, RAM memory 8 GB DDR3-1333 and SO Microsoft^®^ Windows 7 Enterprise) with MATLAB 2015a suite. The tests that obtained data from a real scenario (shown in Section 5.2 and Section 5.3) have been also performed using a self-developed prototype system, composed of two boards. The first one has been totally designed by our research team (Figure 8a) to include the critical and sensitive aspects required by our system: the PSD sensor, the conditioning and amplification of nano-amper current signals received from the PSD—very noise sensitive—and digitalization of the four signals provided; the developed board has been connected to a second board with a SoC (system-on-chip), which has a logical area for reconfigurable hardware (a MicroZed™ board based on the Xilinx Zynq^®^-7000). Thus, the second board is configured with the required hardware to obtain the digital signals from the first board and implement a preprocessing block in hardware to later obtain via software the coordinates of the 3D positioning. This flexible structure makes possible to develop future add-ons for better use of data while making easier the design of a higher level application on top of the ARM standard processing system included in the Zynq (Figure 8b). Figure 8c shows the connection of the boards. In Figure 8d, we present the final enclosed system, ready to be used in real scenarios.

### 5.1. Quantification of Error in the Angle of Arrival

The first test is focused in retrieving the error in the determination of the AoA. This is done using an automated workbench (Figure 7) that has 5${}^{\circ }$ of freedom (displacements along three translation axes and rotation around two angular axes), but, in this test, it is not necessary to make use of all of them. In the case of translation movement, the workbench is equipped with magnetic encoders with a 1 mm resolution, and in the case of rotation, the encoders have a resolution of 0.025${}^{\circ }$.

The rotation and movement of the workbench is controlled from a computer (e.g., 5${}^{\circ }$ increments) and the workbench returns once the order is fulfilled which are the real degrees rotated with an error of the resolution of its encoders (in this case).

**Figure 9:**
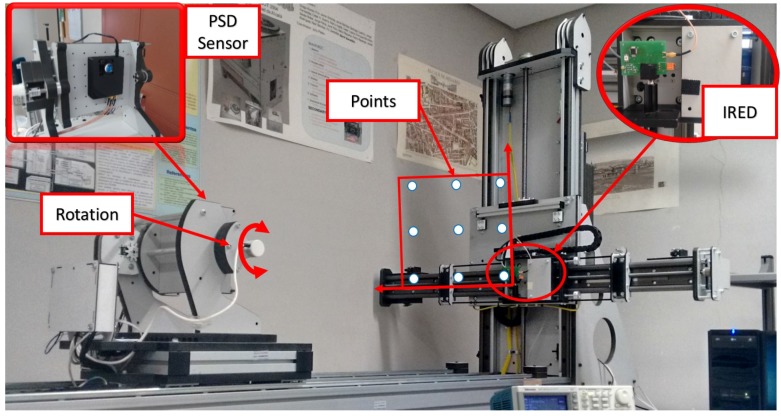
Test for quantification of error in the angle of arrival.

As the error has a circular symmetry around the normal vector to the PSD sensor surface in three-dimensions, to quantify the error done in the determination of the angle of arrival (AoA), only one angle has been modified in the emitter-receiver system (orientation of the detector) while moving the emitter in two-axes along a plane parallel to the original PSD surface (nine different mapped locations), as shown in Figure 9.

Given the accuracy of the automated workbench (0.025${}^{\circ }$), we will be able to determine, with the tolerance given by these values, the error in the PSD positioning.

To perform the experiment, the emitter has been placed in nine different positions forming a planar grid (Figure 9). For each of these nine points, the detector has been placed at the reset location (0${}^{\circ }$), and it has been rotated 5 and 10${}^{\circ }$. The AoA has been calculated from the received signal in the detector for each one of those angular increments. Ideally, in all cases, an increment of 5${}^{\circ }$ should be obtained. Table 3 shows the error for each 5${}^{\circ }$ increment, the differences between the angles measured by the receiver for the different orientations.

This test has been carried out averaging 100 measurements captured for each point; the system is configured with a sampling frequency of 10 MS/s; the emitted signal is a sinusoidal signal (511 cycles of a 50 kHz frequency). The sinusoidal signal has been captured with the PC-based system obtaining the RMS value of the received signal.

**Figure 10:**
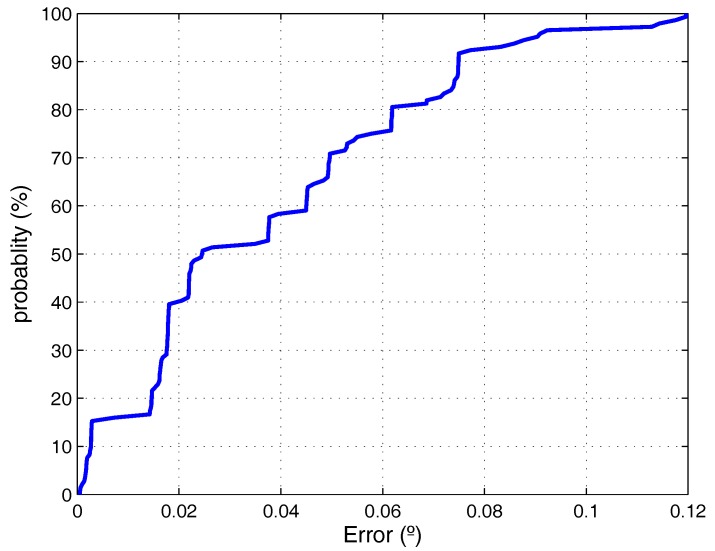
Cumulative Distribution Function (CDF) representation of the obtained errors considering all the points and measurements included in Table 3.

Figure 10 shows the Cumulative Distribution Function (CDF) obtained considering all the available measurements; it can be seen that, in 97% of the measures, the error is less than 0.1°.

The resolution of the workbench magnetic sensor is 0.025${}^{\circ }$ while rotating the detector orientation in the empirical tests. However, it is worth noting that, to accurately perform the movements required in the tests, the workbench sensors resolution is more than enough. The uncertainty (maximum error) in the determination of the rotation angles is about the order of magnitude of the given encoder resolution. Therefore, the measurements will be affected by the encoder resolution, but, in any case, the test helps us to know that the measurements can be obtained with that accuracy.

### 5.2. Static Experimental Tests

**Figure 11:**
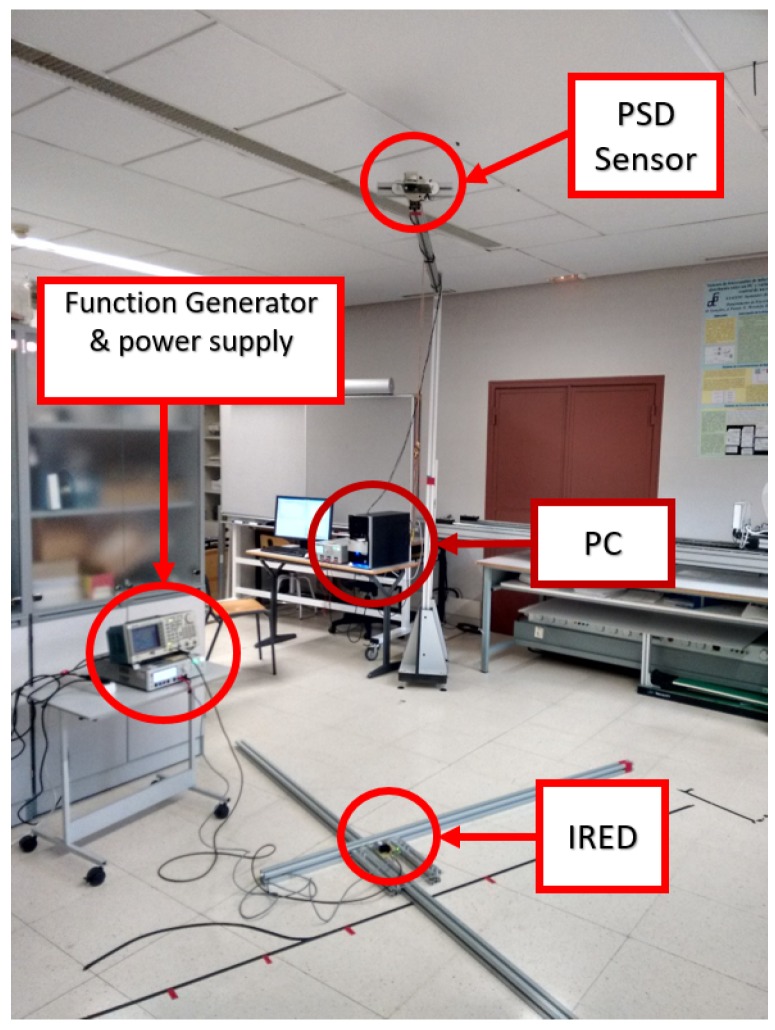
Environment where the static tests were performed.

In this section, we report the static tests performed in the environment shown in Figure 11. The first step was to calibrate the plane, which entailed moving the mobile agent.

The height between the receiver and the plane was 2.5 m, thus covering an area of approximately 1.3 × 1.3 m${}^{2}$. One of the tests was to describe a circumference with a radius of 500 ± 1 mm, comparing this with the radius value obtained from the measurements to quantify the error.

#### 5.2.1. Calibration of the Plane

**Figure 12:**
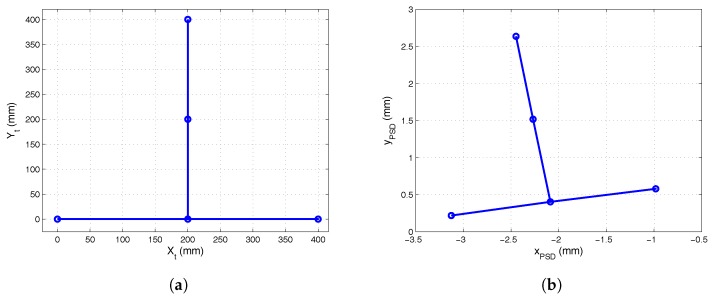
(**a**) calibration template for plane calibration; (**b**) template points in the image plane of the PSD sensor.

To calibrate the plane along which the mobile robot would move, we used a five LED calibration template such as that shown in Figure 12a. This template was placed on the plane of movement to obtain the extrinsic parameters with respect to the PSD sensor, and the parameters of the plane of movement of the mobile robot were obtained from the 3D coordinates of the template LEDs, as indicated in Section 3.1. The projection of these points on the image plane is shown in Figure 12b. The template points and the image plane points were used to obtain the values for the rotation matrix and translation vector parameters shown in (18) and (19), respectively:

(18) $$R=\left(\begin{array}{ccc} 0.9201 & -0.1617 & 03667\\ 0.1291 & 0.9867 & 0.0462\\ -0.3697 & 0.0148 & 0.9288 \end{array}\right),$$

(19) $$T=\left(\begin{array}{c} -564.3\\ 83.288\\ 2575.8 \end{array}\right).$$

**Figure 13:**
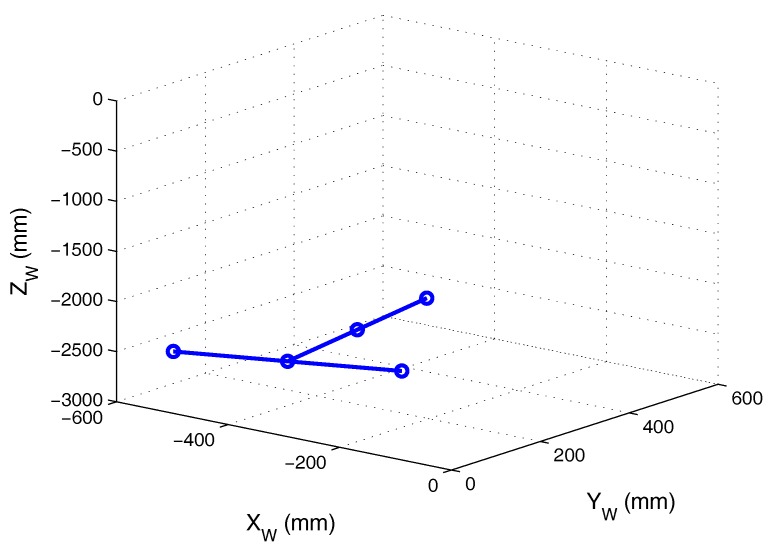
Template points in the environment.

Once the rotation matrix and translation vector had been obtained, they were used to calculate the 3D coordinates of the template points with respect to the PSD receiver. Table 4 shows these coordinates and Figure 13 shows their projection onto the environment.

Lastly, we calculated the plane parameters, which are shown in Table 5:

#### 5.2.2. Determination of the 3D Position

Experiments in Section 5.2.2 have been carried out with the following setup: a total of 100 measurements are captured for each point for later averaging; the system is configured with a sampling frequency of 10 MS/s in this case.

When a sinusoidal signal is used, the receiver captures 511 cycles of a 50 kHz frequency sinusoidal signal to obtain its rms value. When GOLD sequences are used, the correlation peak value is calculated from 511 chirp sequences (also at a frequency of 50 kHz). These tests have been done using the PC-based platform previously defined.

**Figure 14:**
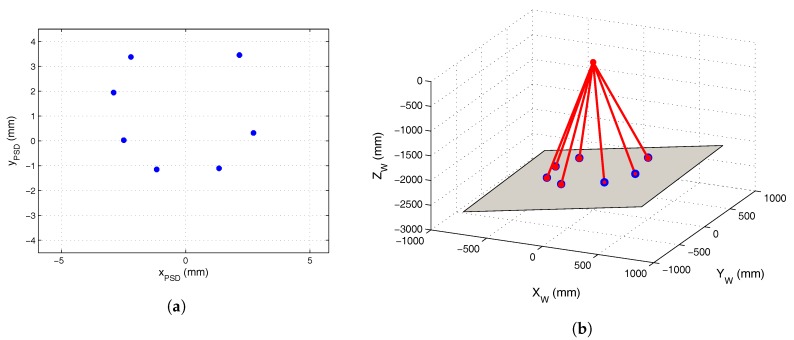
(**a**) points on the circumference in the image plane of the receiver; (**b**) projection of the points in the image plane onto the plane.

With the transmitter placed describing a circumference of 500 ± 1 mm, we obtained images of these points in the sensor plane and calculated the angle of arrival. The 3D coordinates of the positions were obtained from the intersection of the lines given by the angles of incidence and the plane. Figure 14a shows the points calculated in the image plane of the receiver and Figure 14b shows the projection of the points onto the plane. Because the plane was not coplanar to the receiver, it was necessary to change the reference to the plane of movement in order to measure the radius and compare this with the real radius.

**Figure 15:**
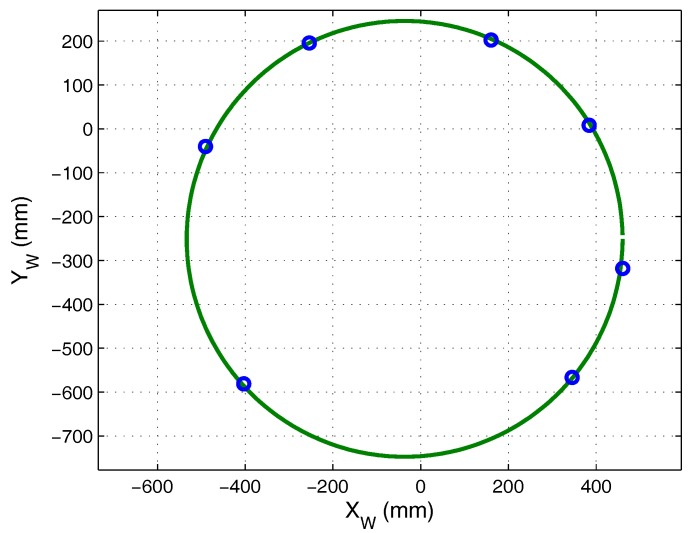
Circumference that best fits the points projected onto the plane.

In Figure 15, the reference system points on the plane of movement are shown in blue, and the circumference that best fits these points is shown in green, where: the radius of the circumference was 496.644 mm, the RMSE (Root Mean Square Error) was 2.2089 mm and the greatest error was 3.4272 mm.

The radius value obtained was similar to that of the circumference, with an error of 3.3560 ± 1 mm, indicating that, in a static case, this method is highly accurate. Furthermore, these results also indicate that the parameters of the plane of movement contained little error.

In the previous test, the coverage area is restricted by the lens system FoV with a focal lenght of *f* = 16 mm having a FoV around 29°. This FoV leads to a coverage area of 1.3 × 1.3 m${}^{2}$ from a height of 2.5 m. We would require a lens with a shorter focal length to obtain a larger area coverage. Because the area of the sensor is 10 × 10 mm${}^{2}$, the lens diameter should be about one inch. However, currently, a one inch lens with a focal length *f*< 16 mm is not commercially available in the market, thus not allowing to make those tests. To enlarge the coverage area, we can increase the height. The geometric error will increase linearly with the distance, as a function of the incidence angle and SNR of the signals.

Regarding the accuracy error, the worst case to be considered is the impact on points in the periphery of the PSD sensor, with a large angle of incidence in the FoV. Take into account that we have considered sensor points to not be more distant from the sensor center than ±4.5 mm. Therefore, Table 6 shows the errors in the determination of the location of a mobile agent that corresponds with the most distant impact point (4.5 mm, 4.5 mm) for different heights and different SNR, with the surface of the PSD sensor coplanar to the surface of the agent movement. The results were obtained from an average of 100 trial tests considering introducing a white Gaussian noise $\left(N\left(0,\sigma^{2}\right)\right)$ with several SNRs: 20 dB, 30 dB and 40 dB, considering a working frequency of 50 kHz, sampling frequency of 10 MS/s and an acquisition time of 10.2 ms (equivalent to capture 511 signal cycles).

As it could be expected, the error varies linearly with the height and is very small when the SNR > 30 dB.

This test was repeated using a GOLD sequence instead of a 50 kHz tone. With a sequence length greater than 127 symbols, the results were similar to those indicated, fitting perfectly for sequences of 511 cycles.

Table 7 shows the processing time using both a PC based system and FPGA (Field Programmable Gate Array) based system.

It is worth noting that, working with FPGA, we could assume that acquisition time and processing time are overlapped, and it is important to note that, for this test, 200 samples per cycle/chirp have been used. As it can be deduced from the obtained results, acquisition time (10.2 ms capturing 511 periods or sequence chirps) determines measurements per second. As it is deduced, the number of measurements reaches the value of 100 per second. If the emission frequency is increased or the number of cycles or samples per cycle reduced, the number of measurements per second increases proportionally.

### 5.3. Dynamic Tests

Lastly, we conducted two dynamic tests, one using a test bench in order to have a ground truth and a second test using the system in a real environment. The first test was done to determine the accuracy that can be achieved with the system, since the automated test bench data is accurately known (ground truth) and the second test was done to compare the results with artificial vision systems (developed also by our research group and based on Kinect cameras). From those tests, we can conclude that the results are similar, the PSD sensor being a cheaper system that is also simple and fast.

In the first case captured, we have taken 81 points forming a grid of 20 × 20 cm${}^{2}$,with a distance between emitter and receiver of 1 m and moving the emitter at a constant speed of 0.2 m/s. These tests were performed using two different types of signals. In one case, sinusoidal signals have been emitted and the position has been calculated from the RMS value of the signals obtained from the PSD sensor (50 kHz and 10.2 ms of acquisition time, which are equivalent to 511 periods). In the other case, GOLD sequences (of 511 chirps) have been emitted, obtaining the impact point from the peak values of the correlation of the PSD signals.

**Figure 16:**
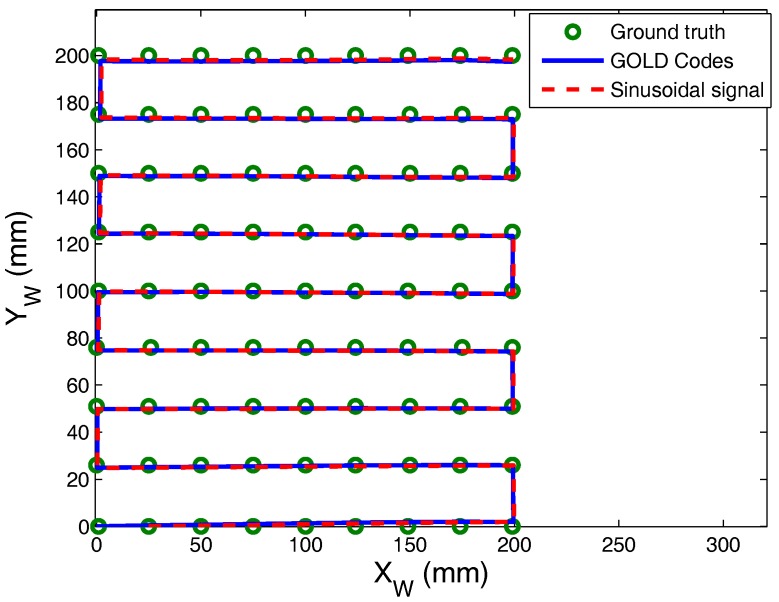
Results of the dynamic test.

The error results are shown graphically in Figure 16 and quantified in Table 8. It can be seen that the system yields a highly accurate determination of the location for both types of emissions (considering a SNR ≥ 40 dB). As it has been previously noted, the error will increase linearly along larger distances.

About making real tests in an environment with obstacles, these would have two main effects. First, if the obstacles blind the LOS (Line-of-Sight) path between emitter and detector, the positioning will not be possible. Second, considering that there is an LOS path, then the obstacles will introduce an MP effect in the determination of the location. An in-depth analysis is out of the scope of our paper (the MP effect will be studied in future works). However, in our related work [33], we have verified the effect of MP in our system and the result was that the fact of having obstacles that generate MP has a very little effect on the determination of the impact point with this technique. Note that the RSS received at each electrode of the PSD sensor is a subtractive composition of currents, which partially compensates the MP and their effect in the location determination.

To test the performance of the gold-code correlation working in noisy environments or with low SNR, the same test done in the test before was performed with other SNR values (the original test considered a 30 dB SNR), retrieving the error done in the positioning for the 81 points under analysis.

**Figure 17:**
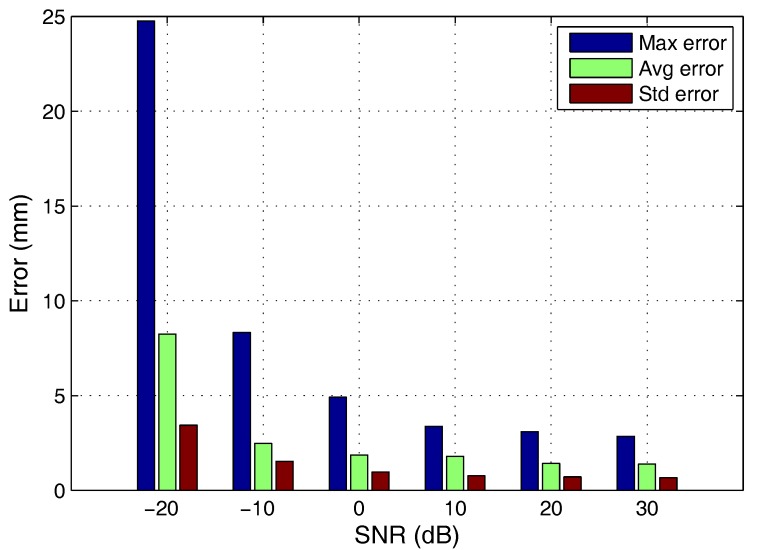
Positioning error when emitting GOLD-code sequences for different SNR values.

From this test, the maximum error, the average error and the standard deviation varying the SNR from −20 to 30 dB values, with 1 m of the distance, sequence length 511 and 10 MHz sampling frequency, has been verified. Table 9 shows the numerical results obtained and Figure 17 shows these data in graphical form. It can be verified that the performance of the system is quite good as long as the SNR does not go below −10 dB.

**Figure 18:**
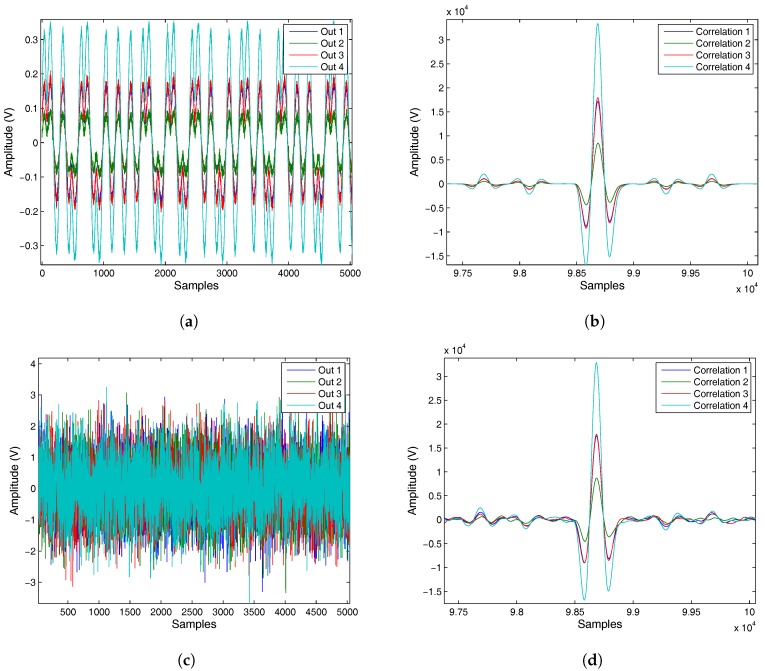
(**a**) Out signals of the PSD sensor with 30 dB of SNR; (**b**) correlations of the Out signals with 30 dB; (**c**) Out signals of the PSD sensor with−10 dB of SNR; (**d**) correlations of the Out signals with 30 dB.

Figure 18 shows two examples of the signals detected by the PSD sensor and later correlation considering 30 dB of SNR (Figure 18a,b) and −10 dB of SNR (Figure 18c,d). Figure 18a,c show the voltage signals (codes) obtained from the PSD sensor. Figure 18b,d shows their correlations after processing.

**Figure 19:**
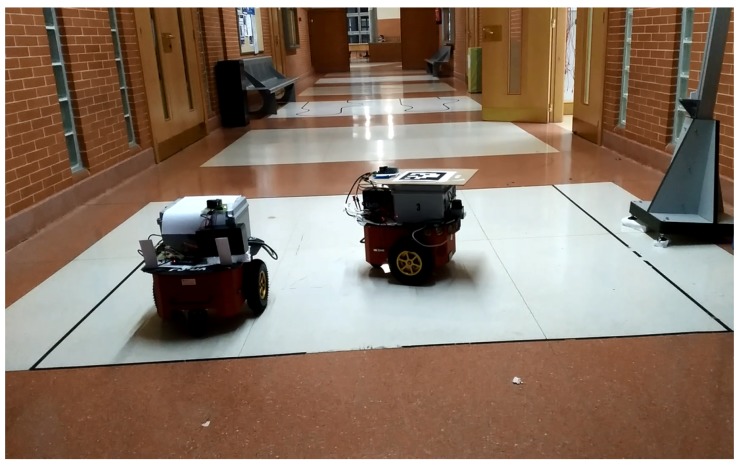
Environment where the dynamic tests were performed.

The second test was conducted in the environment shown in Figure 19, where the P3DX robot can be seen. The receiver was located 4 m above the plane of movement, and the parameters obtained in plane determination are shown in Table 10.

As in the previous cases, the transmitter used a 50 kHz signal (with an acquisition time for position calculation of 10 ms) in one test, and a GOLD sequence of 1023 chirps in the other. The sampling frequency was 10 MHz. The environment where the test was performed was equipped with another vision-based IPS using Kinect cameras, and this was used to compare the position detected by each system. To perform the test, the robot was programmed to move in a spiral. In the first test, positioning was performed by our system (using a 50 kHz emitted tone) and was Kinect IPS based; in the second test, the spiral movement was repeated and positioning was performed using GOLD sequences.

**Figure 20:**
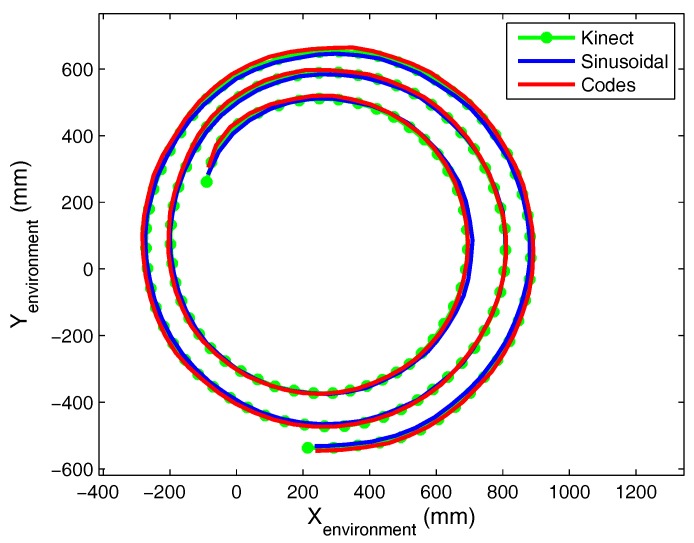
Positioning results obtained in dynamic tests in a real environment.

In Figure 20, the paths obtained using the three systems (Kinect and the proposed method with 50 kHz tones and the GOLD sequence) have been superimposed. The mean difference between the Kinect and proposed positioning systems using a tone was 3.6172 cm (STD = 2.4863 cm), while the maximum difference was 15.8692 cm. A comparison of the results obtained with our system using a tone and a sequence indicated that the maximum difference was 1.4 cm.

A video of real-time positioning using the Kinect and the proposed system with a 50 kHz tone can be viewed in [34]. The full video can be viewed on the web.

**Figure 21:**
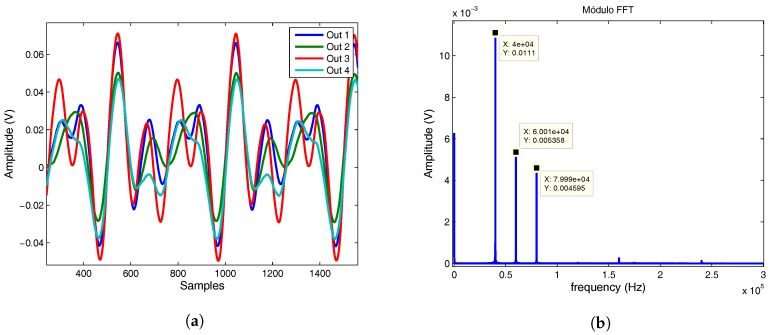
(**a**) Out signals of the PSD sensor; (**b**) Fast Fourier Transformation (FFT) of the signal 4.

To show the behavior of the proposed system working simultaneously with several mobile agents, a multi-agent test has been setup composed of three emitters using each one a different frequency (40 kHz, 60 kHz and 80 kHz). The emitters were placed on a right triangle with 400 mm legs and the PSD sensor was placed at a height of 2.7 m. The output signals from the PSD system are shown in Figure 21a (the four signals are a composition of the three emitted frequencies). Figure 21b shows the FFT (Fast Fourier Transformation) of the signal (Out 4) where the three frequency components are obtained, besides the offset.

**Figure 22:**
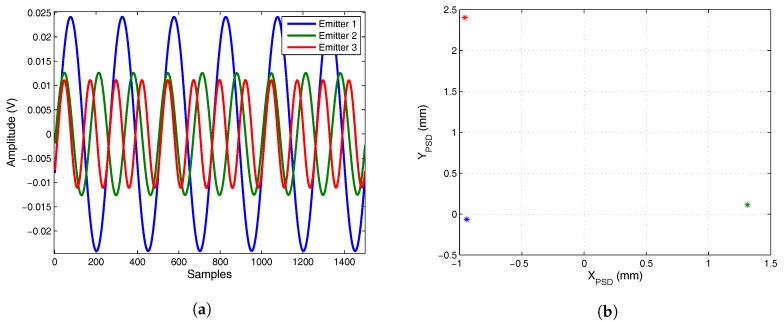
(**a**) filtered signal; (**b**) impact points of the three InfraRed Emitter Diode sensors (IREDs) on PSD sensor.

The signals are further processed by three band pass filters centered on the working frequencies corresponding to each emitter, obtaining the three output signals shown in Figure 22a. Performing this filtering for all the signals and applying Equations (1) and (2), the impact points of the IREDs are obtained (shown in Figure 22b).

**Figure 23:**
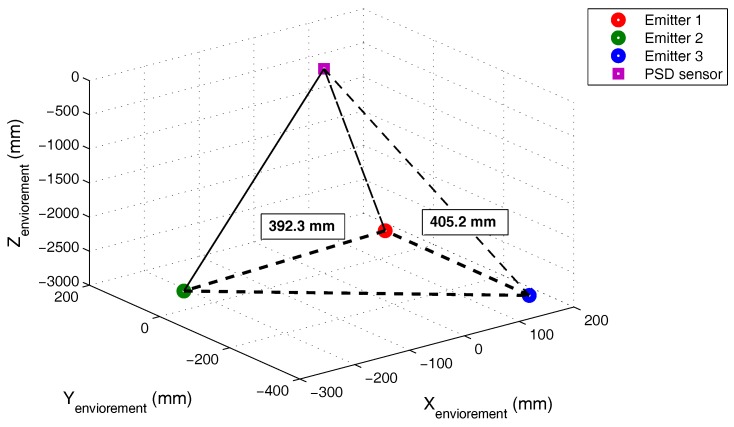
3D positioning of the three IREDs.

Computing the AoA and knowing the plane of movement for the emitter agents, their 3D positions are determined (shown in Figure 23). The errors in the determination of the right triangle legs (ideally 400 mm) are 7.7 mm and 5.2 mm.

### 5.4. Results Comparison

There are few research works for IPS using PSD sensors, but more using optical signals by means of other technologies. The comparison with other research works have a limited relative value; however, in Table 11, we show the technology used, coverage area and accuracy of different related IPS systems compared with ours.

## 6. Conclusions

We have presented a proposal here for an IPS based on PSD sensors that yields millimetric accuracies, as has been demonstrated in various experimental tests. The positioning system and method have been designed so that more agents can easily be added (a question that is being addressed in current studies) without affecting accuracy. In addition, more receivers can be added, whether working together or not, because they do not need to be synchronized. We have proposed two types of transmissions, one using frequency tones and another using different sequences. We have analyzed the sensitivity of the system to error, indicating how this affects measurements and the precautions to take to ensure that the system is accurate in all cases. With regard to accuracy, experimental tests have demonstrated that the system commits errors of less than 0.1${}^{\circ }$ in the 97% of the measurements performed when determining angles and the RMSE in determining the position, with PSD placed on a height of 2.5 m, was 2.2089 mm and the greatest error was 3.4272 mm. The proposed method does not entail a high computational load because it is sufficient to measure the angle of arrival and calculate the intersection of the lines given by the angle and the plane to determine position. In future works we will develop a system for determining multi agents’ positions using sequences and the self-developed prototype (shown in Figure 8c). In addition, we will analyze the effects of MP in our method and develop a new positioning method based on RSS and AoA.

## Figures and Tables

**Table 1:** Coordinates of the points of the calibration template for the plane.

	$P_{1}$	$P_{2}$	$P_{3}$	$P_{4}$	$P_{5}$	$P_{6}$	$P_{7}$	$P_{8}$
$X_{t}$	0	750	1500	1500	1500	750	0	0
$Y_{t}$	0	0	0	750	1500	1500	1500	750

**Table 2:** Parameters of the planes used.

	A	B	C	D
Coplanar plane	0 mm	0 mm	1 mm	−4000 mm
Inclined plane	0	−1 mm	3.7321 mm	−14,928.20 mm

**Table 3:** Calculation error in the angle of arrival.

Error (${}^{\circ }$)	Rotation Angle = 5°			Rotation Angle = 10°		
	Max	Avg	Std	Max	Avg	Std
$P_{1}$	0.046	0.045	0.000	0.042	0.027	0.005
$P_{2}$	0.089	0.087	0.001	0.039	0.036	0.001
$P_{3}$	0.014	0.013	0.000	0.127	0.093	0.001
$P_{4}$	0.032	0.018	0.007	0.086	0.089	0.004
$P_{5}$	0.090	0.089	0.001	0.101	0.094	0.000
$P_{6}$	0.088	0.088	0.000	0.080	0.076	0.001
$P_{7}$	0.102	0.098	0.003	0.061	0.042	0.004
$P_{8}$	0.109	0.107	0.000	0.095	0.092	0.000
$P_{9}$	0.0097	0.0095	0.001	0.084	0.078	0.001

**Table 4:** 3D coordinates of the template points.

	$P_{1}$	$P_{2}$	$P_{3}$	$P_{4}$	$P_{5}$
*X* (mm)	−564.3005	−380.2726	196.2447	−412.6149	−444.9571
*Y* (mm)	83.2886	109.1055	134.9224	306.4510	503.7964
*Y* (mm)	2575.8	2501.9	2427.9	2504.8	2507.8

**Table 5:** Values obtained for the plane parameters.

A	B	C	D
−58,672.84	−7388.56	−148,608.18	350,291,748.99

**Table 6:** Error for different heights and SNRs.

Height/		4 m			4.5 m			5 m		
Usable Coverage		2.250 × 2.250 m${}^{2}$			2540 × 2540 m${}^{2}$			2.810 × 2.810 m${}^{2}$		
**3D Coordinates of Point under Analysis**		**(1125, 1125, 4000)**			**(1270, 1270, 4500)**			**(1405, 1405, 5000)**		
	Error (mm)	Max	Avg	Std	Max	Avg	Std	Max	Avg	Std
SNR (dB)										
20		61.13	20.31	10.66	72.48	23.08	12.02	76.42	25.39	13.33
30		21.14	6.57	3.47	22.53	7.23	3.73	24.61	8.20	4.29
40		8.98	2.60	1.36	9.17	2.63	1.23	9.34	2.60	1.39

**Table 7:** Comparison of processing time.

Processing Time (ms)	Sinusoidal Signal	GOLD Sequences
PC based system + Matlab	20.154	40.103
Developed prototype + FPGA	0.039	8.028

**Table 8:** Errors of the dynamic test.

Errors (mm)	Average	Maximum	Standard Deviation
Sinusoidal signal	1.30	2.28	0.49
GOLD codes	1.37	2.66	0.61

**Table 9:** Positioning error when emitting GOLD-code sequences for different SNR values.

	Error (mm)	Maximum	Average	Standard Deviation
SNR (dB)				
−20		24.763	8.250	3.436
−10		8.336	2.471	1.526
0		4.921	1.863	0.971
10		3.380	1.789	0.768
20		3.098	1.425	0.711
30		2.854	1.398	0.679

**Table 10:** Plane parameter values for dynamic testing in a real environment.

A	B	C	D
−62,362.88	−291,179.63	520,104.61	−2,080,258,774.40

**Table 11:** Comparison of results obtained by systems proposed from other research works.

Research Work	Technology Used	Coverage Area	Accuracy
Proposed method	IRED emitter + PSD sensor	1 × 1 × 2.5 m${}^{3}$	<0.5 cm
Park [18]	IRED emiter + PSD sensor	3 × 3 × 2.5 cm${}^{3}$	2–9 cm
Lin [24]	3 LED light+ photodetector	20 × 20 × 15 cm${}^{3}$	2 cm
Zachár [25]	3 LED beacons + camera	15 × 30 × 3 m${}^{3}$	10 cm
Kumaki [26]	4 LED arrays + camera	0.3 × 0.3 × 1 m${}^{3}$	5 cm
Sakai [27]	IRED Beacon +12 photodetector	7 × 2 × 2.3 m${}^{3}$	70 cm
Huynh [20]	VLC + Image sensor+ acelerometer	7 × 7 × 3.5 m${}^{3}$	10 cm
Nakazawa [22]	4 LED light + Image sensor	5.4 × 7.5 × 3 m${}^{3}$	10 cm

## Abbreviations

The following abbreviations are used in this manuscript:

AoA	Angle of Arrival
BPSK	Binary Phase Shift Keying
BW	Bandwidth
DFT	Discrete Fourier Transformation
FFT	Fast Fourier Transformation
FoV	Field of View
IIR	Infinite Impulse Response
IPS	Indoor Positioning System
IRED	Infrared Emitting Diodes
IS	Image Sensor
LED	Light Emitting Diode
LOS	Line of Sight
MP	Multipath
NIR	Near-Infrared
PSD	Position Sensitive Device
RMS	Root Mean Square
RMSE	Root Mean Square Error
RSS	Received Signal Strength
SNR	Signal-to-Noise Ratio
STD	Standard Deviation
SVD	Singular Value Decomposition
TDOA	Time Difference of Arrival
VLC	Visible Light Communication
